# Causal relationship between immune factors and Guillain-Barré syndrome: A Mendelian randomization study

**DOI:** 10.1097/MD.0000000000050444

**Published:** 2026-08-28

**Authors:** Taichuan Xu, Xingguo Zhang, Xianfa Xu, Wentao Xiao, Wenjie Li, Xian Zhang

**Affiliations:** a Department of Orthopedics, Nanjing University of Chinese Medicine, Nanjing, Jiangsu, China; b Department of Spine, Wuxi Affiliated Hospital of Nanjing University of Chinese Medicine, Wuxi, Jiangsu, China.

**Keywords:** causal inference, Guillain-Barré syndrome, immune traits, inflammatory proteins, Mendelian randomization

## Abstract

Guillain-Barré syndrome (GBS) is an immune-mediated polyneurogenic disorder that is strongly correlated with immune factors. However, causal relationships between these factors remain unclear. We used 2-sample Mendelian randomization (MR) and inverse MR analysis. Immune factors were used as the outcome for GBS exposure. Cochrane *Q* test was used to detect heterogeneity. Simultaneously, the MR-Egger intercept test and MR-Pleiotropy Residual Sum and Outlier tests were used to check for horizontal pleiotropy. MR Steiger was used to estimate the direction of causal inference when the instrumental variables were insufficient in inverse MR. Eighteen immune traits and 1 inflammatory protein were identified as causally involved in the risk of GBS. Reverse MR revealed that GBS was correlated with the levels of eleven immune traits. The sensitivity analyses did not identify any potential heterogeneity or horizontal pleiotropy. Steiger did not find any evidence of reverse causation. We used 2-sample MR to investigate the causal relationship between immune factors and GBS, and our results provide clues for further research.

## 1. Introduction

Guillain-Barré syndrome (GBS) is an immune-mediated inflammatory polyradiculoneuropathy, primarily characterized by progressive symmetrical limb weakness with decreased or absent reflexes and sensory deficits. It is currently considered to be the most common cause of acute flaccid paralysis.^[1]^ GBS includes several variants such as acute inflammatory demyelinating polyneuropathy, acute motor axonal neuropathy, Miller Fisher syndrome, and Bickerstaff brainstem encephalitis.^[1]^ Approximately 10,000 people worldwide develop GBS each year, and population-based studies in North America and Europe suggest an incidence of 0.81 to 1.91 cases per 1,00,000 people per year.^[2]^ Approximately 14% of patients with GBS will develop severe permanent disability within the first year; a higher proportion (54–89%) will experience pain, and the majority will suffer from chronic fatigue.^[3-5]^

Immune factors are thought to be involved in the progression of GBS.^[6]^ Imbalances in T helper 1 (Th1), Th2, Th17, regulatory T (Treg) cells, and in classically activated macrophages (M1) and alternatively activated macrophages (M2) are observed in GBS and experimental autoimmune neuritis (EAN).^[6]^ It has been demonstrated that elevation of Th1 cytokines may be linked to the initial stages of GBS progression, which is characterized by neuronal inflammation. Conversely, augmentation of immune responses associated with Th2 has been shown to facilitate subsequent recovery from GBS.^[7]^ An increased number of circulating Th22 cells has been observed to correlate with disease severity in patients with GBS, irrespective of the specific GBS subtype. Th17 and Th22 cells in the acute phase of GBS express a range of proinflammatory mediators, including IL-17, IL-22, and TNF-α. These factors contribute to the augmentation of the inflammatory and autoimmune responses, ultimately leading to GBS progression.^[8]^ The attack of gangliosides on Schwann cells and axons is associated with the presence of macrophages, T cells, and B cells.^[9]^ In addition, Asbury et al discovered that substantial lymphocyte infiltration into the nerves is a common feature in patients with GBS and that persistent inflammation may be associated with relapse.^[10]^ However, conclusions regarding the relationship between certain immune traits and inflammatory proteins and the risk of GBS remain inconsistent. Serum interferon-*γ* (IFN-*γ*) levels have been reported to be significantly elevated in patients with GBS, positively correlated with clinical disability, and significantly decreased after treatment with intravenous immunoglobulin (IVIg).^[11]^ Another study found that CD4+ CD25− T cells in peripheral blood were converted to CD4+ CD25+ T cells induced by IFN-*γ*,^[12]^ whereas the lack of IFN-*γ* exacerbated EAN by up-regulating Th17 cells.^[13]^ Considering some controversial findings and the bias in traditional observational studies, further assessment of the causal relationship between GBS and immune factors is necessary.^[14-16]^

GBS is a rare disease, and observational studies are often susceptible to limitations, such as insufficient cases, design bias, confounders, reverse causal interference, and time and money. Mendelian randomization (MR) is an epidemiological research method that uses genetic variation as instrumental variables (IVs) to infer causal relationships between exposure and outcome.^[17]^ Since alleles are randomly assigned and determined as they are passed from parent to offspring, confounding factors and reverse causality can be minimized.^[18]^ MR offers a distinct advantage in immune-mediated diseases such as GBS, where traditional observational studies are particularly prone to reverse causation. The immune system changes dynamically with disease state, making it difficult to determine whether an observed immune alteration drives pathology or merely reflects it.^[19,20]^ In this study, we used bidirectional MR analysis to investigate the causal relationships between immune traits, inflammatory proteins, and GBS. We anticipate that through the application of MR analysis, we can offer valuable insights into the pathogenesis and potential therapeutic avenues for GBS.^[21]^

## 2. Materials and methods

### 2.1. Study design

We performed a 2-sample bidirectional MR to explore the causal relationship between GBS, 731 immune traits, and 91 inflammatory proteins. In the forward direction, immune traits were treated as exposures and GBS as the outcome. In the reverse direction, GBS was treated as the exposure and each immune trait as the outcome. Our MR study followed the STROBE-MR guidelines.^[22]^ The checklist is summarized in Table S1, Supplemental Digital Content 1.

### 2.2. Data source

The data for the 731 immune trait characteristics came from an interrogation of whole-genome sequencing of approximately 22 million genetic variants in 3757 Sardinians reported by Valeria Orrù et al.^[23]^ This includes 4 trait types (382 median fluorescence intensities for surface antigens, 192 relative cell counts, 118 absolute cell counts, and 32 morphometric parameters) and 7 panels (monocytes, B cells, Tregs, TBNK, maturation stages of T cells, cDC, and myeloid cells) of immune phenotypic traits. Genetic associations for 91 circulating inflammatory proteins were derived from a meta‑analysis of 11 European cohorts totaling 14,824 individuals, measured using the Olink Targeted Inflammation Panel.^[24]^ GBS outcome data were acquired from the Finnish Genetic (FinnGen) Consortium, which contains genetic data on disease endpoints for more than 5,00,000 Finns. We chose the summary statistics of GBS based on the G6_GUILBAR dataset published by the FinnGen Consortium in 2023, which includes 4,05,581 European pedigrees (nCase = 445, nControl = 4,05,136) with 19,34,55,539 single nucleotide polymorphisms (SNPs).^[25]^ All data were obtained from publicly available GWAS consortia and were based on individuals of European descent, which minimizes population stratification bias.

### 2.3. Selection of instrumental variables

For the forward MR analysis, we applied a genome-wide significance threshold of *P* < 5 × 10^−8^ with clumping parameters *r*^2^ = 0.001 and window size = 10,000 kb to ensure that the IVs were strongly associated with the exposure. For the reverse MR analysis, we adopted a more lenient threshold of *P* < 5 × 10^−6^ to ensure a sufficient number of IVs, as GBS has a relatively small effective sample size and stricter thresholds would yield too few instruments for reliable estimation. This strategy is commonly adopted in bidirectional MR studies when the outcome is a rare disease.^[26]^ To evaluate instrument strength, we calculated the *F*-statistic for each SNP using the formula *F* = (*R*^2^(N−K−1))/(K(1−*R*^2^)), where *R*^2^ represents the proportion of exposure variance explained by each SNP, N is the sample size of the exposure GWAS, and K is the number of SNPs in the instrumental variable. SNPs with an *F*-statistic < 10 were considered weak instruments that may introduce finite-sample bias and were excluded from subsequent analyses.

### 2.4. Mendelian randomization analysis and sensitivity analysis

The inverse-variance weighted (IVW) results obtained by meta-analyzing the Wald ratios estimated for each SNP provided relatively accurate causal estimates in the absence of horizontal pleiotropy. Therefore, IVW is the primary method.^[27]^ To address potential pleiotropy, we also applied 3 robust methods. The weighted median, weighted mode, and MR-Egger methods are robust to horizontal pleiotropy, provide more reliable causal estimates in a wider range of situations, and are often used as complementary methods to IVW.^[28]^ Weighted median provides a consistent estimate if at least 50% of the weight comes from valid IVs.^[29]^ MR‑Egger regression allows for an intercept term that detects directional pleiotropy and provides a causal estimate corrected for such bias.^[30]^ Weighted mode estimates the causal effect based on the most common pleiotropy‑free subset of IVs. We report results from all 4 methods but base our primary inference on the IVW estimate, with the other methods serving as sensitivity checks. Our MR analysis was conducted as an exploratory study,^[31]^ and it was considered nominally significant when *P* < .05.^[32]^ We did not apply a stringent multiple‑testing correction such as Bonferroni, as this would drastically inflate false‑negatives and mask potentially important signals. To ensure the direction of causality, we performed inverse MR. Steiger test was applied in the reverse direction (when IVs were limited) to confirm the direction of causality, ensuring that the observed association was not due to reverse causation from the outcome to the exposure.^[33]^ Cochran IVW *Q* test was used to detect heterogeneity in IVs. A significant *Q* statistic (*P* < .05) would indicate substantial heterogeneity among the SNP‑specific Wald ratios, which could weaken the reliability of the IVW estimate. The MR-Egger intercept test and MR Pleiotropy Residual Sum and Outlier tests were used to evaluate potential horizontal pleiotropic effects. Leave-one-out sensitivity analysis was used to ascertain whether the causal effects were attributable to particular SNPs. Statistical analyses were performed using R software (version 4.3.0), RMediation package (version 1.2.2), and TwoSampleMR package (version 0.5.6).

## 3. Results

### 3.1. The causal relationship between immune traits and Guillain-Barré syndrome

**Figure 1.**
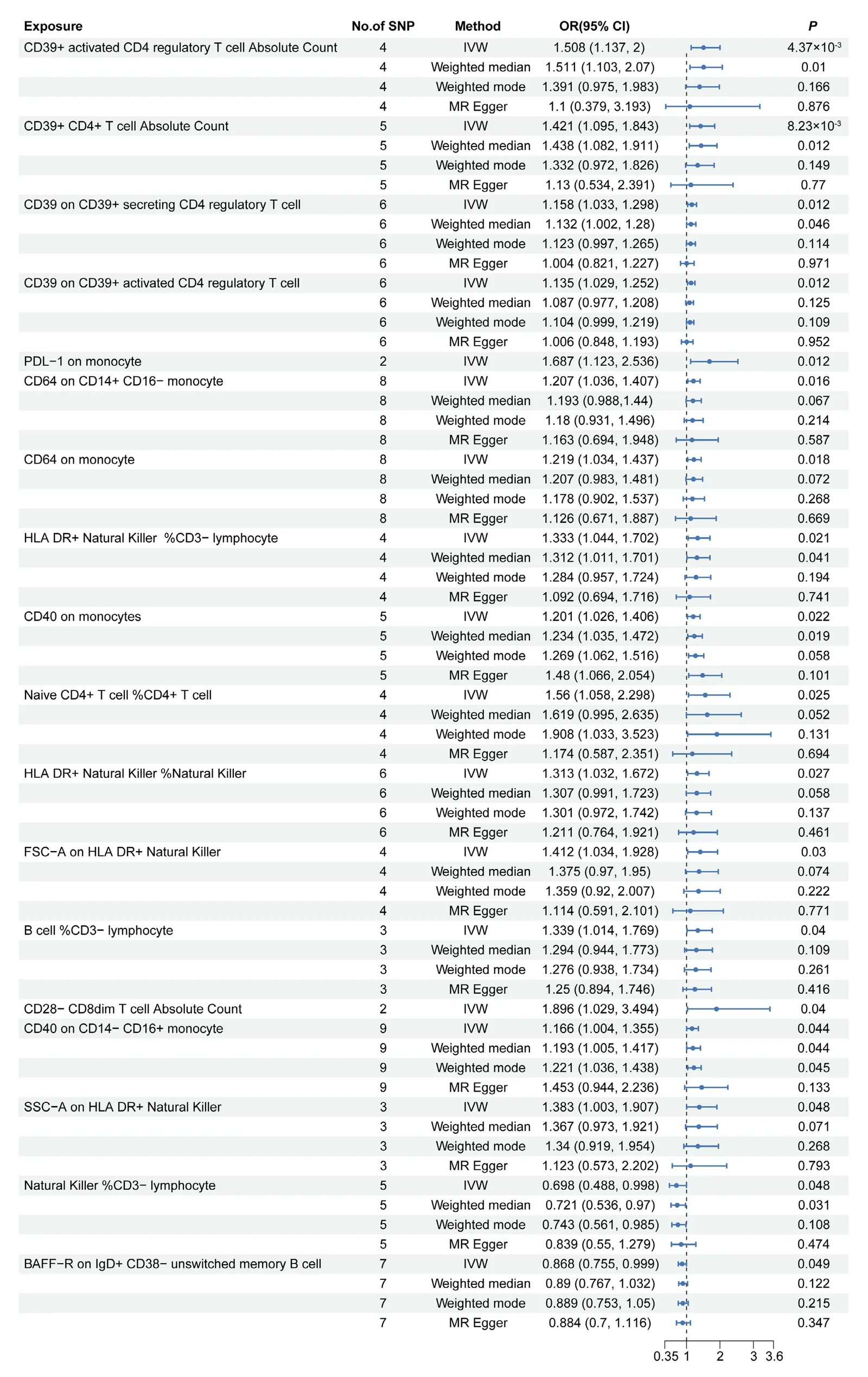
Forest plot of nominally significant causal effects for 18 immune traits on GBS. CI = confidence interval, GBS = Guillain-Barré syndrome, IVW = inverse-variance weighted, MR = Mendelian randomization, OR = odds ratio.

**Figure 2.**
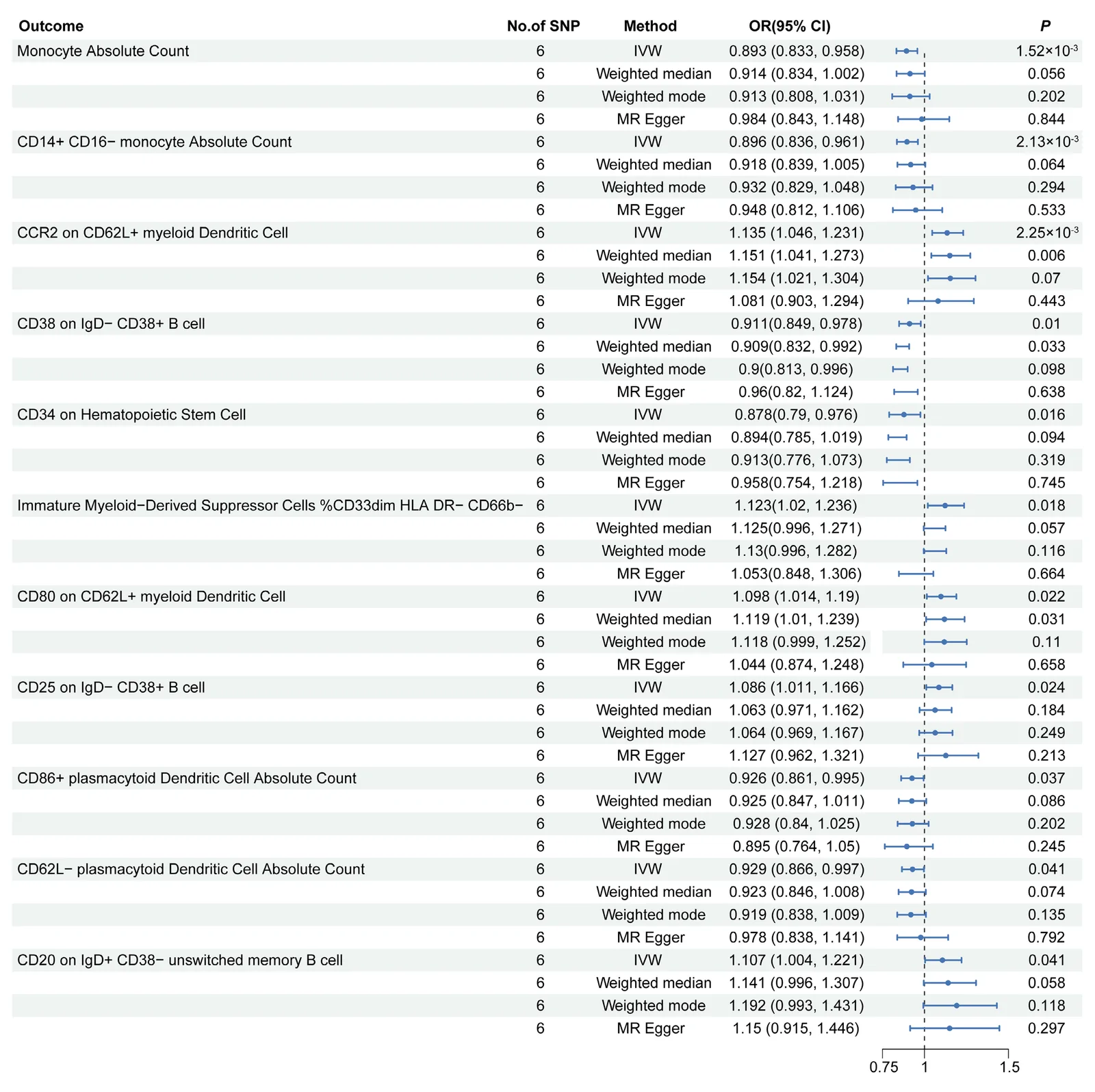
Forest plot of nominally significant causal effects for 11 GBS on immune traits. CI = confidence interval, GBS = Guillain-Barré syndrome, IVW = inverse-variance weighted, MR = Mendelian randomization, OR = odds ratio.

The MR results suggested that 18 immune traits were associated with GBS. Among these immune traits, elevated levels of sixteen immune traits were associated with a high risk of GBS (OR > 1, *P* < .05), of which 1 belonged to the Maturation stages of T cell panel (Naive CD4+ T cell %CD4+ T cell), 5 to the Monocyte panel (PDL-1 on monocyte, CD64 on CD14+ CD16- monocyte, CD64 on monocyte, CD40 on monocytes, CD40 on CD14− CD16+ monocyte), 5 to the TBNK panel (HLA DR+ Natural Killer %CD3− lymphocyte, HLA DR+ Natural Killer %Natural Killer, FSC-A on HLA DR+ Natural Killer, B cell %CD3− lymphocyte, SSC-A on HLA DR+ Natural Killer), and 5 to the Treg panel (CD39+ activated CD4 regulatory T cell Absolute Count, CD39+ CD4+ T cell Absolute Count, CD39 on CD39+ secreting CD4 regulatory T cell, CD39 on CD39+ activated CD4 regulatory T cell, CD28- CD8dim T cell Absolute Count), while the other 2 immune traits were detected as potential protective factors for GBS (OR < 1, *P* < .05), of which 1 belonged to B cell panel (BAFF-R on IgD+ CD38- unswitched memory B cell) and 1 belonged to TBNK panel (Natural Killer %CD3− lymphocyte). More details on the results of the causal inference are presented in Table S2, Supplemental Digital Content 2, and information on the IVs and results of the *F*-statistic are presented in Table S3, Supplemental Digital Content 3. No significant heterogeneity or horizontal pleiotropy was found, as shown in Table S2, Supplemental Digital Content 2. Figure 1 shows the forest plot; the scatter plot is shown in Figure S1, Supplemental Digital Content 4, and the funnel plot is seen in Figure S2, Supplemental Digital Content 5. Plots of the leave-one-out analysis are presented in Figure S3, Supplemental Digital Content 6 and show no potential effect of SNPs on causality. After evaluating the causal effects of GBS on immune traits, we found that GBS was associated with decreased levels of 6 immune traits (OR < 1, *P* < .05) and increased levels of 5 immune traits (OR > 1, *P* < .05). Of the 6 immune traits with decreased levels, 1 belonged to the B-cell panel (CD38 on IgD− CD38+ B cells), 2 to the cDC panel (CD86+ plasmacytoid dendritic cell absolute count, CD62L− plasmacytoid dendritic cell absolute count), 2 to the monocyte panel (monocyte absolute count, CD14+ CD16− monocyte absolute count), and 1 to the myeloid cell panel (CD34 on hematopoietic stem cells). Of the 5 immune traits with elevated levels, 2 belonged to the B cell panel (CD25 on IgD− CD38+ B cells and CD20 on IgD+ CD38− unswitched memory B cells), 2 belonged to the cDC panel (CCR2 on CD62L+ myeloid dendritic cells and CD80 on CD62L+ myeloid dendritic cells), and 1 belonged to the myeloid cell panel (immature myeloid-derived suppressor cells %CD33dim HLA DR− CD66b−). No heterogeneity or horizontal pleiotropy was observed in this study. The results of the MR and sensitivity analyses are presented in Table S4, Supplemental Digital Content 7, and those of the IVs and *F*-statistics are displayed in Table S5, Supplemental Digital Content 8. The results of the forest plot are shown in Figure 2, and the scatter plot, funnel plot, and leave-one-out analysis are shown in Figures S4, S5 and S6, Supplemental Digital Content 9, respectively.

### 3.2. The causal relationship between inflammatory proteins and Guillain-Barré syndrome

**Figure 3.**
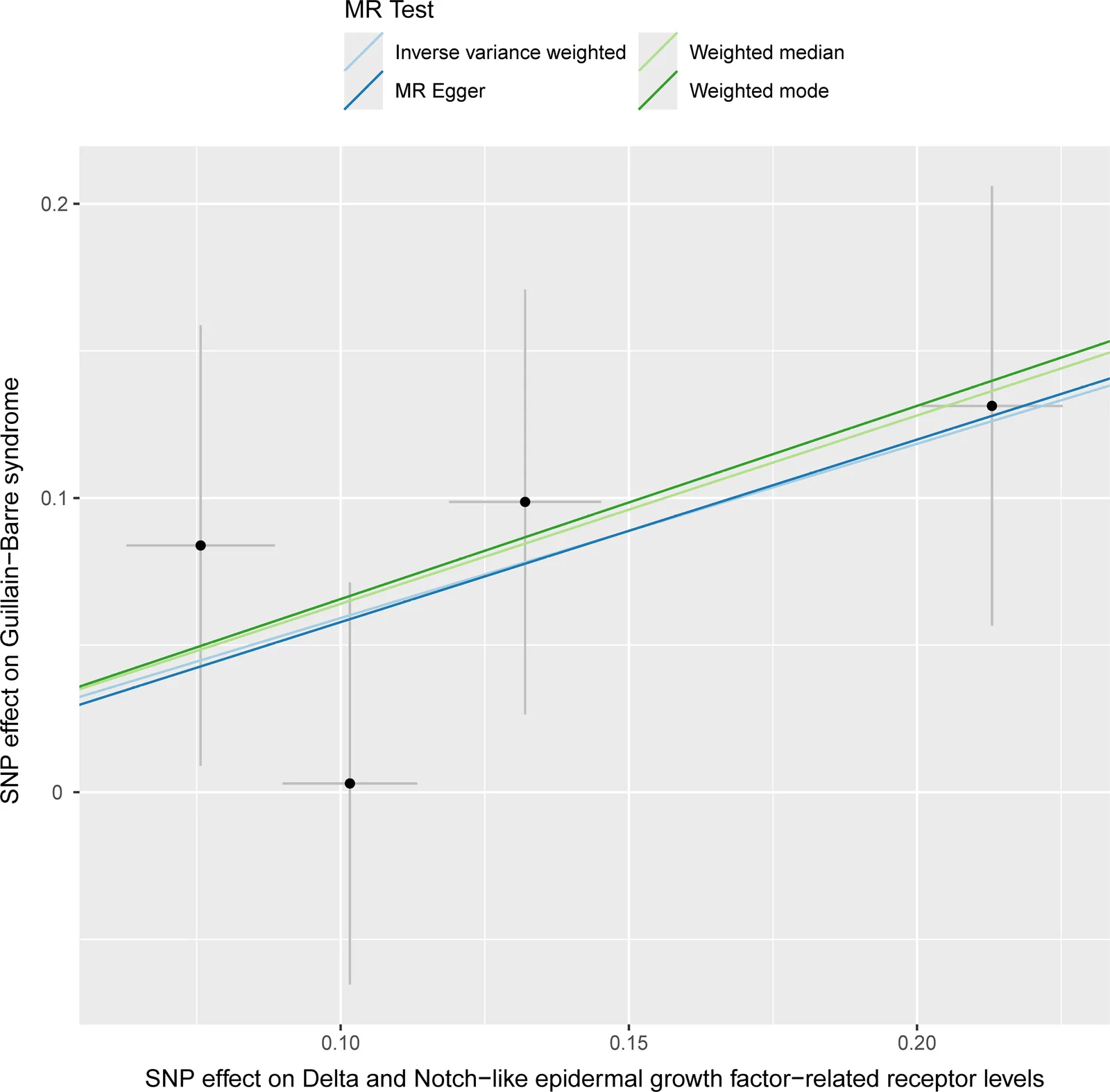
Scatter plot for MR analysis of DNER levels on GBS. DNER = Delta and Notch-like epidermal growth factor-related receptor, GBS = Guillain-Barré syndrome, MR = Mendelian randomization, SNPs = single nucleotide polymorphisms.

The results showed that higher levels of Delta and Notch-like epidermal growth factor-related receptors (DNER) are associated with a higher risk of GBS. Neither horizontal pleiotropy nor heterogeneity was observed. Steiger found no evidence of reverse causation. The scatter plot is shown in Figure 3. The funnel plot and leave-one-out analysis results are shown in Figure S7, Supplemental Digital Content 10. The results of the MR and sensitivity analyses are listed in Table S6, Supplemental Digital Content 11. Details of the MR Steiger, IVs, and their strengths are listed in Table S7, Supplemental Digital Content 12. However, no causal effect of GBS on inflammatory proteins was found in reverse MR analysis.

## 4. Discussion

We assessed the causal relationship between GBS and 731 immune traits and 91 inflammatory traits with GBS using MR analysis based on publicly available GWAS summary data. Eighteen immune traits were found to exert a causal effect on GBS, and GBS was found to be associated with 11 immune traits. DNER was found to be a risk factor for GBS. However, no potential causal effect of GBS on inflammatory proteins has been identified.

Intravenous Immunoglobulin (IVIg) and plasma exchange (PE) are considered equally effective treatments for improving GBS prognosis.^[34]^ IVIg is thought to inhibit Fc-mediated activation of immune traits, neutralize anti-ganglioside antibodies, and downregulate autoantibody-mediated complement activation. PE may be achieved by eliminating neurotoxic antibodies, complement factors, and other humoral inflammatory mediators during GBS treatment.^[35]^ However, IVIg and PE do not appear to modulate immunity through a specific pathway, and approximately 25% of patients with GBS still require intensive care units for respiratory failure or autonomic dysfunction after treatment, 9% to 17% are severely disabled or die, and a significant proportion of patients present with prolonged fatigue and pain.^[36-38]^ Specific biomarkers that can ultimately aid in the clinical diagnosis and monitoring of disease progression, as well as biological targets with favorable therapeutic effects in GBS, are still lacking.^[39]^ A combination of clinical features, cerebrospinal fluid (CSF) analysis, and nerve conduction studies (NCS) is frequently employed in the diagnosis of GBS. However, NCS and CSF tests have low sensitivity in the early stages of GBS, and the diversity of clinical presentations may lead to erroneous diagnoses.^[35]^ A deeper understanding of the immune mechanisms involved in GBS is crucial for improving its diagnosis, treatment, and prognosis.^[40]^

### 4.1. CD39+ Tregs and their potential role in GBS pathogenesis

The present study identified a causal relationship between the presence of CD39 as a surface marker and GBS development. CD39, or ectonucleoside triphosphate diphosphohydrolase 1, is an integral membrane protein belonging to the ENTPDase family that is thought to catalyze the phosphohydrolysis of extracellular ATP and ADP released under conditions of inflammatory stress and cell injury.^[41]^ In T cell populations, CD39 is mainly expressed in the CD4+ T cell and Treg subsets.^[42]^ Currently, there are conflicting observational studies on CD39 expression in neurological disorders.^[41]^ Several studies have reported a decrease in the number of CD39+ Treg cells or no significant difference in their percentage in patients with relapsing-remitting multiple sclerosis (RRMS) and a decrease in their number in stable multiple sclerosis (MS).^[43-45]^ However, increased CD39 expression and CD39+ Treg ratio have been reported in the peripheral blood mononuclear cells of MS patients, and CD39 expression is also increased in the cerebrospinal fluid of RRMS patients.^[46-48]^ Álvarez-Sánchez suggested that CD39 activity in PBMC and increased CD39 expression in Tregs during RRMS exacerbations may be compensatory mechanisms to counteract some of the dysfunction of Treg cells in patients with MS; however, a possible pro-inflammatory role of CD39 cannot be excluded.^[47]^ Similarly, CD39 was found to have increased expression in lymphocytes from patients with systemic lupus erythematosus,^[49]^ and in CD39−deficient mice, 2,4,6-trinitrobenzene sulfonic acid-induced colitis was attenuated.^[50]^ In humans, CD39 expression on Tregs is particularly high at sites of inflammation, and a higher proportion of CD39+ Tregs express CCR4, CCR6, and CXCR3, which are involved in T-cell migration to sites of inflammation.^[51]^ These studies support our conclusions to some extent; however, further studies are needed to explore the role of different CD4+ T cells and CD4+ Treg cells expressing CD39 in the onset and progression of GBS, as well as their potential as biomarkers for the diagnosis and treatment of GBS.

### 4.2. Monocyte subpopulations and their causal association with GBS

Our findings underscore the intricate causal relationship between monocytes and GBS infection. The results of our MR analysis indicated that genetically predicted elevated levels of PDL-1 on monocytes, CD64 on CD14+ CD16 − monocytes, CD64 on monocytes, CD40 on monocytes, and CD40 on CD14− CD16+ monocytes were associated with an increased risk of GBS. However, reverse MR showed that GBS resulted in decreased monocyte absolute count and CD14+ CD16− monocyte absolute count. This finding suggests that a more cautious interpretation of the relationship between monocytes and GBS is necessary. Some researchers believe that monocytes play a pivotal role in GBS, with the transformation of monocytes correlating with the severity of GBS.^[52]^ A study by Li et al revealed that the absolute monocyte count was elevated in patients with GBS compared with that in healthy controls. Furthermore, there was a positive correlation between absolute monocyte count and disease severity. Logistic regression analysis demonstrated that this correlation is an independent predictor of GBS.^[53]^ This finding is consistent with our results. Notably, different monocyte subpopulations have different functional and phenotypic switching potential. Further studies are required to elucidate these mechanisms. Chuluundorj et al posited that activated monocytes are among the earliest immune traits to reach the brain and trigger inflammation in MS.^[54]^ They characterized the total number of monocytes and subpopulations in vitro in 29 patients with MS and 20 healthy controls and found that the levels of CD40, HLA-DR, CD64, CD86, and CCR2 were significantly elevated in total monocytes and were most pronounced in CD16+ monocytes. In vitro stimulation with lipopolysaccharide demonstrated an increase in the production of HLA-DR, CD64, and CD86 in monocytes from patients with MS.^[46]^ Using the expression of markers in human monocyte-derived M1 and M2 macrophages, Vogel found that M1 markers such as CD64, CD40, and CD86 were abundantly expressed in macrophages and activated microglia in active demyelinating MS lesions.^[55]^ In addition, IgM anti-CD64 antibodies were found in patients with long-term stable MS and were associated with lower relapse rates.^[56]^

### 4.3. NK cells and HLA-DR surface markers in the context of neuroinflammation

Our study identified natural killer cells and HLA-DR+ surface markers with a causal effect on GBS. Emerging evidence suggests that natural killer (NK) cells are involved in the development and progression of neuroimmune diseases such as GBS and are quickly becoming a target of choice for the treatment of neuroimmune diseases.^[57]^ Lum et al concluded that ZIKV infection abnormally activates NK cells and promotes the release of IFN-γ and CD107a, which may induce severe neurological complications, such as GBS.^[58]^ HLA-DR is an MHC class II molecule that is highly expressed in antigen-presenting cells.^[59]^ In GBS, the activation of circulating macrophages and T lymphocytes is evidenced by enhanced expression of histocompatibility antigens, and several genetic studies have found an association between HLA/HLA-DR and GBS.^[60-62]^ NK cell activation usually results in the appearance of HLA-DR on the cell surface, and NK cells expressing HLA-DR typically exhibit higher proliferation, cytokine production, and cytotoxicity.^[63]^ More evidence suggests that the percentage of HLA-DR-expressing NK cells in the blood and tissues increases under conditions associated with chronic inflammation and various autoimmune diseases.^[57,64]^

### 4.4. DNER as a novel risk factor for GBS

Our study also found that elevated DNER levels are a risk factor for GBS. DNER, an epidermal growth factor-like repeat-sequence single-transmembrane protein, was previously found to be specifically expressed in the dendrites and cell bodies of CNS neurons.^[65]^ The human DNER gene is localized to chromosome 2q37, and some causative mutations leading to neurogenetic disorders have been identified. However, detailed linkage mapping is still needed for validation.^[66,67]^

### 4.5. Clinical relevance

Our findings carry several clinical implications. First, the identified immune trait subsets, particularly CD39+ Tregs, CD40+ and CD64+ monocytes, and HLA-DR+ NK cells, may serve as candidate peripheral biomarkers for risk stratification or early diagnosis of GBS, though this requires prospective validation.^[68]^ Second, these markers represent potential therapeutic targets. For instance, modulating CD39 activity or blocking CD40/CD64-mediated monocyte activation could be explored as adjunctive strategies to current immunotherapies.^[69]^ Third, the bidirectional associations we observed suggest that GBS itself induces secondary immune alterations, implying that the timing of immunomodulatory intervention may be critical.^[29]^ Early treatment might not only control acute inflammation but also prevent maladaptive immune feedback. Finally, our MR-based framework demonstrates the utility of genetic approaches for prioritizing immune targets in rare diseases, which may inform future trial design.^[70]^ These hypotheses warrant further experimental and clinical investigation.^[30]^

### 4.6. Limitations

Our study has some limitations. First, it should be noted that, constrained by available GWAS data, the study population was limited to individuals of European ancestry. While this homogeneous ancestry reduces population stratification bias, it also limits the generalizability of our findings to other ethnic groups. Future studies with multi-ethnic GWAS data are warranted to validate our findings across diverse populations. Second, because GBS is a rare disease and current summary-level data do not provide more specific information on GBS subtypes such as acute inflammatory demyelinating polyneuropathy and acute motor axonal neuropathy, future stratified analyses based on updated GWAS data on GBS subtypes are warranted. Third, although we employed multiple sensitivity analyses to detect and correct for horizontal pleiotropy, we cannot entirely exclude the possibility of unmeasured pleiotropic effects, which may bias the results. Fourth, the use of a more lenient *P*-value threshold (*P* < 5 × 10^−6^) in the reverse MR analysis, while necessary to obtain sufficient IVs, may increase the risk of including weak instruments, although all included SNPs had *F*-statistics > 10. Finally, as an exploratory MR study, we did not apply strict multiple testing correction, and our findings should be considered hypothesis-generating rather than confirmatory. Future studies with larger sample sizes and more detailed phenotypic data are needed to validate our findings.

## 5. Conclusions

We comprehensively and systematically explored the causal relationship between immune factors and GBS using MR analysis. These results may provide insights that could inform further research into the etiology and pathogenesis of GBS, as well as the development of prevention and treatment strategies.

## Author contributions

**Conceptualization:** Taichuan Xu, Wentao Xiao, Xian Zhang.

**Data curation:** Taichuan Xu, Xianfa Xu.

**Formal analysis:** Taichuan Xu, Wentao Xiao.

**Investigation:** Xianfa Xu, Wentao Xiao, Wenjie Li.

**Methodology:** Taichuan Xu.

**Project administration:** Xian Zhang.

**Resources:** Xingguo Zhang, Xian Zhang.

**Software:** Taichuan Xu, Wentao Xiao.

**Supervision:** Xingguo Zhang, Xianfa Xu, Xian Zhang.

**Validation:** Xianfa Xu, Wenjie Li, Xian Zhang.

**Visualization:** Wentao Xiao, Wenjie Li.

**Writing – original draft:** Taichuan Xu, Xingguo Zhang.

**Writing – review & editing:** Taichuan Xu, Xingguo Zhang, Xianfa Xu, Xian Zhang.




























