# Supplementary material for: Causal relationship between immune factors and Guillain-Barré syndrome: A Mendelian randomization study
Source: Medicine (Baltimore). 2026 Aug 28;105(35):e50444. doi: 10.1097/MD.0000000000050444 (PMC13529122; doi:10.1097/MD.0000000000050444)
Supplement: Supplementary file 1 [file medi-105-e50444-s001.docx]

**Table S1 STROBE-MR checklist of recommended items to address in reports of Mendelian randomization studies**^1^ ^2^

| **Item No.** | **Section** | **Checklist item** | **Page No.** | **Relevant text from manuscript** |
| --- | --- | --- | --- | --- |
| 1 | **TITLE and ABSTRACT** | Indicate Mendelian randomization (MR) as the study’s design in the title and/or the abstract if that is a main purpose of the study | 1-2 | Title: “Causal relationship between immune factors and Guillain‑Barré syndrome: a Mendelian randomization study”. Abstract: “We used two-sample Mendelian randomization (MR) and inverse MR analysis.” |
|  | **INTRODUCTION** |  |  |  |
| 2 | **Background** | Explain the scientific background and rationale for the reported study. What is the exposure? Is a potential causal relationship between exposure and outcome plausible? Justify why MR is a helpful method to address the study question | 2-4 | “MR is an epidemiological research method that uses genetic variation as instrumental variables… confounding factors and reverse causality can be minimized.” Also cites inconsistent observational findings and the dynamic nature of immune system in GBS. |
| 3 | **Objectives** | State specific objectives clearly, including pre-specified causal hypotheses (if any). State that MR is a method that, under specific assumptions, intends to estimate causal effects | 3-4 | “In this study, we used bidirectional MR analysis to investigate the causal relationships between immune traits, inflammatory proteins, and GBS.” (No pre‑registered hypotheses stated.) |
|  | **METHODS** |  |  |  |
| 4 | **Study design and data sources** | Present key elements of the study design early in the article. Consider including a table listing sources of data for all phases of the study. For each data source contributing to the analysis, describe the following: |  |  |
|  | a) | Setting: Describe the study design and the underlying population, if possible. Describe the setting, locations, and relevant dates, including periods of recruitment, exposure, follow-up, and data collection, when available. | 4 | “All data were obtained from publicly available GWAS consortia and were based on individuals of European descent…” Dates not explicitly given, but references to 2023 FinnGen release. |
|  | b) | Participants: Give the eligibility criteria, and the sources and methods of selection of participants. Report the sample size, and whether any power or sample size calculations were carried out prior to the main analysis | 4 | Immune traits: 3,757 Sardinians; inflammatory proteins: 14,824 Europeans; GBS: 405,581 Finns (445 cases, 405,136 controls). No power calculation reported. |
|  | c) | Describe measurement, quality control and selection of genetic variants | 4 | “genome‑wide significance threshold of P < 5×10⁻⁸ with clumping r² = 0.001 and window = 10,000 kb” for forward; reverse used P < 5×10⁻⁶. F‑statistic > 10 used. |
|  | d) | For each exposure, outcome, and other relevant variables, describe methods of assessment and diagnostic criteria for diseases | 4 | Immune traits measured as MFI, cell counts, etc.; inflammatory proteins by Olink panel; GBS defined by FinnGen’s G6_GUILBAR dataset (ICD codes implied). |
|  | e) | Provide details of ethics committee approval and participant informed consent, if relevant | 12 | “The data for this study were obtained from publicly available GWAS data and do not require additional ethical approval.” (Original studies had their own ethics.) |
| 5 | **Assumptions** | Explicitly state the three core IV assumptions for the main analysis (relevance, independence and exclusion restriction) as well assumptions for any additional or sensitivity analysis | 4-5 | Relevance ensured by strong SNP‑exposure associations (F>10); independence/exclusion addressed via sensitivity analyses (MR‑Egger intercept, MR‑PRESSO) to detect pleiotropy |
| 6 | **Statistical methods: main analysis** | Describe statistical methods and statistics used |  |  |
|  | a) | Describe how quantitative variables were handled in the analyses (i.e., scale, units, model) | 5-6 | Odds ratios (OR) per standard deviation change; no covariate adjustment in two‑sample MR because summary data used. |
|  | b) | Describe how genetic variants were handled in the analyses and, if applicable, how their weights were selected | 4-5 | Clumping and F‑statistic selection; IVs were independent SNPs; no weighting beyond the MR methods. |
|  | c) | Describe the MR estimator (e.g. two-stage least squares, Wald ratio) and related statistics. Detail the included covariates and, in case of two-sample MR, whether the same covariate set was used for adjustment in the two samples | 5-6 | “IVW results obtained by meta‑analyzing the Wald ratios”; no covariates because two‑sample MR using summary statistics. |
|  | d) | Explain how missing data were addressed | Not applicable | No missing data handling mentioned; GWAS summary data typically have no missingness after QC. |
|  | e) | If applicable, indicate how multiple testing was addressed | 5-6 | “We did not apply a stringent multiple‑testing correction such as Bonferroni, as this would drastically inflate false‑negatives considered nominally significant when P < 0.05.” |
| 7 | **Assessment of assumptions** | Describe any methods or prior knowledge used to assess the assumptions or justify their validity | 5-6 | Heterogeneity (Cochrane’s Q), horizontal pleiotropy (MR‑Egger intercept, MR‑PRESSO), and Steiger test for reverse causation were used. |
| 8 | **Sensitivity analyses and additional analyses** | Describe any sensitivity analyses or additional analyses performed (e.g. comparison of effect estimates from different approaches, independent replication, bias analytic techniques, validation of instruments, simulations) | 5-6 | Leave‑one‑out analysis, MR‑PRESSO, weighted median, weighted mode, MR‑Egger, and reverse MR with Steiger test. |
| 9 | **Software and pre-registration** |  |  |  |
|  | a) | Name statistical software and package(s), including version and settings used | 5-6 | “R software (version 4.3.0), RMediation package (version 1.2.2), and TwoSampleMR package (version 0.5.6).” |
|  | b) | State whether the study protocol and details were pre-registered (as well as when and where) | Not applicable | No mention of pre‑registration; likely not pre‑registered. |
|  | **RESULTS** |  |  |  |
| 10 | **Descriptive data** |  |  |  |
|  | a) | Report the numbers of individuals at each stage of included studies and reasons for exclusion. Consider use of a flow diagram | 4 | Numbers given for each GWAS (e.g., 3,757, 14,824, 405,581); no exclusions described because summary data used. |
|  | b) | Report summary statistics for phenotypic exposure(s), outcome(s), and other relevant variables (e.g. means, SDs, proportions) | 4 | Provides sample sizes and case/control numbers; no means/SDs for immune traits (GWAS summary). |
|  | c) | If the data sources include meta-analyses of previous studies, provide the assessments of heterogeneity across these studies | Not applicable | No meta‑analysis of previous studies; data are from single GWAS consortia. |
|  | d) | For two-sample MR:  i.  Provide justification of the similarity of the genetic variant-exposure associations between the exposure and outcome samples  ii.  Provide information on the number of individuals who overlap between the exposure and outcome studies | 4 | “All data… based on individuals of European descent, which minimises population stratification bias.” Overlap not quantified, but all are European ancestry. |
| 11 | **Main results** |  |  |  |
|  | a) | Report the associations between genetic variant and exposure, and between genetic variant and outcome, preferably on an interpretable scale | Tables S3, S5, S7 | IVs and F‑statistics are provided in supplementary tables; not in main text but referenced. |
|  | b) | Report MR estimates of the relationship between exposure and outcome, and the measures of uncertainty from the MR analysis, on an interpretable scale, such as odds ratio or relative risk per SD difference | 6-7 | “Eighteen immune traits… associated with GBS (OR > 1, P < 0.05)” and ORs provided in Tables S2, S4, S6. |
|  | c) | If relevant, consider translating estimates of relative risk into absolute risk for a meaningful time period | Not applicable | No absolute risk translation. |
|  | d) | Consider plots to visualize results (e.g. forest plot, scatterplot of associations between genetic variants and outcome versus between genetic variants and exposure) | 6-7 | Figures 1–3 (forest plots, scatter plots) and supplementary figures provided |
| 12 | **Assessment of assumptions** |  |  |  |
|  | a) | Report the assessment of the validity of the assumptions | 6-7 | “No significant heterogeneity or horizontal pleiotropy was found”; details in Tables S2, S4, S6. |
|  | b) | Report any additional statistics (e.g., assessments of heterogeneity across genetic variants, such as *I^2^*, Q statistic or E-value) | 6-7 | Cochrane’s Q and MR‑Egger intercept are reported in tables; no E‑value mentioned. |
| 13 | **Sensitivity analyses and additional analyses** |  |  |  |
|  | a) | Report any sensitivity analyses to assess the robustness of the main results to violations of the assumptions | 6-7 | Leave‑one‑out, MR‑PRESSO, weighted median, weighted mode, MR‑Egger all used and results described. |
|  | b) | Report results from other sensitivity analyses or additional analyses | 6-7 | Reverse MR and Steiger test results reported |
|  | c) | Report any assessment of direction of causal relationship (e.g., bidirectional MR) | 6-7 | Reverse MR performed; Steiger test applied (in inverse MR) to confirm direction. |
|  | d) | When relevant, report and compare with estimates from non-MR analyses | Not applicable | No non‑MR comparisons made. |
|  | e) | Consider additional plots to visualize results (e.g., leave-one-out analyses) | 6-7 | Leave‑one‑out plots provided in supplementary figures (S3, S6, S7B). |
|  | **DISCUSSION** |  |  |  |
| 14 | **Key results** | Summarize key results with reference to study objectives | 7 | “Eighteen immune traits were found to exert a causal effect on GBS, and GBS was found to be associated with 11 immune traits. DNER was found to be a risk factor for GBS.” |
| 15 | **Limitations** | Discuss limitations of the study, taking into account the validity of the IV assumptions, other sources of potential bias, and imprecision. Discuss both direction and magnitude of any potential bias and any efforts to address them | 11 | Lists European‑only population, lack of subtype data, possible pleiotropy, lenient P‑value in reverse MR, exploratory nature without multiple correction. |
| 16 | **Interpretation** |  |  |  |
|  | a) | Meaning: Give a cautious overall interpretation of results in the context of their limitations and in comparison with other studies | 11 | “These results may provide insights… but should be considered hypothesis‑generating rather than confirmatory.” |
|  | b) | Mechanism: Discuss underlying biological mechanisms that could drive a potential causal relationship between the investigated exposure and the outcome, and whether the gene-environment equivalence assumption is reasonable. Use causal language carefully, clarifying that IV estimates may provide causal effects only under certain assumptions | 8-10 | Detailed mechanistic explanations for CD39+ Tregs, monocytes, NK cells, DNER; uses cautious language (“may”, “suggest”). |
|  | c) | Clinical relevance: Discuss whether the results have clinical or public policy relevance, and to what extent they inform effect sizes of possible interventions | 10-11 | Discusses potential biomarkers, therapeutic targets, and timing of intervention; acknowledges need for validation. |
| 17 | **Generalizability** | Discuss the generalizability of the study results (a) to other populations, (b) across other exposure periods/timings, and (c) across other levels of exposure | 11 | “limited to individuals of European ancestry… limits generalizability to other ethnic groups.” |
|  | **OTHER INFORMATION** |  |  |  |
| 18 | **Funding** | Describe sources of funding and the role of funders in the present study and, if applicable, sources of funding for the databases and original study or studies on which the present study is based | 11 | “This study was supported by the Natural Science Foundation of Jiangsu Province (BK20231147).” |
| 19 | **Data and data sharing** | Provide the data used to perform all analyses or report where and how the data can be accessed, and reference these sources in the article. Provide the statistical code needed to reproduce the results in the article, or report whether the code is publicly accessible and if so, where | 11-12 | “All data generated or analyzed during this study are included in this article or in the supplementary material.” Code not explicitly mentioned, but methods provide package names. |
| 20 | **Conflicts of Interest** | All authors should declare all potential conflicts of interest | 11 | “No potential conflict of interest was reported by the author(s).” |

This checklist is copyrighted by the Equator Network under the Creative Commons Attribution 3.0 Unported (CC BY 3.0) license.

1.	Skrivankova VW, Richmond RC, Woolf BAR, Yarmolinsky J, Davies NM, Swanson SA, et al. Strengthening the Reporting of Observational Studies in Epidemiology using Mendelian Randomization (STROBE-MR) Statement. JAMA. 2021;under review.

2.	Skrivankova VW, Richmond RC, Woolf BAR, Davies NM, Swanson SA, VanderWeele TJ, et al. Strengthening the Reporting of Observational Studies in Epidemiology using Mendelian Randomisation (STROBE-MR): Explanation and Elaboration. BMJ. 2021;375:n2233.
